# Induction of Apoptosis by Extract of Persian Gulf Marine Mollusk, *Turbo Coronatus* through the ROS-Mediated Mitochondrial Targeting on Human Epithelial Ovarian Cancer Cells

**Published:** 2019

**Authors:** Fatemeh Zangeneh, Amir Vazirizadeh, Mohammad reza Mirshamsi, Amir Fakhri, Mehrdad Faizi, Jalal Pourahmad

**Affiliations:** a *Department of Pharmacology and Toxicology, School of Pharmacy, Shahid Beheshti University of Medical Sciences, Tehran, Iran. *; b *The Marine Biology and Fishery Science Department, Persian Gulf Institute, Persian Gulf University, Bushehr,Iran.*

**Keywords:** Turbo coronatus, Epithelial ovarian cancer, Gel filtration chromatography, F1 fraction, Apoptosis

## Abstract

Despite recent improvements in treatment, ovarian cancer is still the leading cause of death from gynaecological malignancies. Today, marine mollusks are considered as natural source of new biologically and pharmacologically active compounds by scientists and the pharmaceutical industries. The aim of this study is to investigate the selective apoptotic effects of *Turbo coronatus *crude extract fractions on human epithelial ovarian cancer (EOC) cells and mitochondria. The cells and mitochondria were isolated from cancerous and non-cancerous ovarian tissues and exposed to IC_50 _concentration of F1 fraction for evaluation of mitochondrial and cellular parameters. Our results showed that F1 fraction of *T. coronatus* crude extract significantly induced toxic effects only in the cancerous ovarian mitochondria, including increased reactive oxygen species (ROS) formation, mitochondrial membrane depolarization, mitochondrial swelling, and cytochrome c release.Flow-cytometry analysis demonstrated that F1 fraction of *T. coronatus* progressively induced apoptosis and necrosis only on EOC but not non-cancerous cells. We eventuallyconcluded that F1 fraction of *T. coronatus* crude extract selectively induces apoptosis in EOC through a ROS- mediated pathway.

## Introduction

Amongst gynecologic malignancies ovarian cancer is the second most common and the leading cause of death in women in the U.S.A (1, 2). In 2018, about 22, 240 new cases of ovarian cancer will be detected, and approximately 14,070 women will die due to this cancer in the U.S.A (3). More than 95% of ovarian tumors are diagnosed as epithelial ovarian cancer (EOC) (4), since this cancer is usually diagnosed in the advanced stages due to the lack of specific symptoms. It is fatal and its 5-year survival rate is below 40% (2, 5). Standard treatment for EOC is debulking surgery followed by adjuvant combination chemotherapy. The overall survival rate of advanced EOC is low despite surgery combined with new adjuvant strategies and is approximately 4 months (5). Because none of these approaches can thoroughly protect EOC patients from recurrence and metastasis, new drugs are urgently needed. Recent studies have shown that the induction of apoptosis is resulted from ROS generation and cytochrome-c release into the cytosol causing mitochondrial dysfunction in the ovarian cancer cells (6). Nowadays, targeting apoptosis pathways is one of the mechanisms underlying treatment for many cancers. In several types of cancers, mitochondria undergo a lot of functional and morphological changes (7). One is a remarkable change in cancer cells compared to normal cells in energy metabolism.However, the cancer cells seem to have certain mitochondrial dysfunction due to a variety of factors, such as oncogenic signals, mitochondrial enzyme defects and mtDNA mutations, and thus, rely more on the glycolytic pathway in the cytosol and increase in aerobic glycolysis to generate ATP known as the Warburg effect (8). Since mitochondria play an important role in regulating apoptosis through their effectors, mitochondrial dysfunction in cancer cells may induce more cytochrome-c release, therefore agents that trigger the release of apoptotic factors from mitochondria with minimal side-effects is an approach for cancer treatment. Natural products contain a wide variety of compounds with potent chemotherapeutic or chemopreventive activities. So far, over 60% of the current anticancer drugs are from naturally-derived compounds such as species of plants, animals, marine organisms, and microorganisms (9, 10). Marine environment is an endless source of bioactive compounds with pharmacological and biological properties. The 24 new metabolites reported this year from mollusks is the average number of metabolites usually reported per year for this marine fauna in the past decade (11, 12).

*Turbo coronatus,* common name for the Coronate Moon Turban snail, belongs to the *Turbinidae* family first discovered in Samish Bay, Washington in 1924 (13). It inhabits rockyshores and tidal slits from the Red Sea to South Africa and in the eastern hemisphere from Japan, Korea, Indonesia, and Persian Gulf as well (13, 14).In recent year, several researches have described the pharmacological, immunological, chemical and anti-cancer effects of the Turbo snails extract and fractions (15, 16). Extract and fractions obtained from Turbo snail species were observed to show antineoplastic, acetylcholinesterase inhibition, and cytotoxic effects ref. In this study, crude extract of *T. coronatus* and its fractions were tested for their selective cytotoxic activity on isolated human EOC cells and mitochondria and investigate the underlying cellular and sub cellular mechanisms involved. We demonstrated that crude extract of *T. coronatus* and its fraction caused cytotoxic effects on isolated human EOC cells and mitochondria which were attributed to the crude extract and F1 fraction ­̓s ability to induce apoptosis. The aim of this study is to explore the selective cytotoxic activity of *T. coronatus* crude extract fractions on isolated human epithelial ovarian cancer cells and mitochondria and investigate the underlying cellular and sub cellular mechanisms involved.

## Experimental

*Patients*

The selected patients were recently diagnosed with EOC and aged 40-80 years. They were from the Surgery Center of Valiasr Hospital, Imam Khomeini Hospital Complex, Tehran Medical University, Iran, and had not received any prior radiation or chemotherapy. The solid ovarian cancer samples were collected from areas macroscopically identified as cancer by a pathologist at the time of surgery. Non-cancerous ovarian tissue samples were obtained from sites distant from the tumor and from the same cancerous ovary. This study was approved by the Ethical Committee of Shahid Beheshti University of Medical Sciences. For the present study the informed consent was obtained from all the patients.

*Crude extract preparation*

After the collection of *T. coronatus* from the coastal waters of Bushehr (28.9234° N latitude and 50.8203°E longitude), Iran during summer months, the samples were immediately transported to the laboratory and the viscera were removed by breaking the shells. Briefly, they were homogenized using a homogenizer (IKA, Germany) and homogenate was centrifuged at 12000 × g for 30′. The supernatant was freeze-dried (Christ, UK) and kept at −20 ºC for subsequent experiments (17). 

*Gel filtration chromatography*

600 mg of the crude extract was dissolved in 10 mL of 0.2 M ammonium acetate*.* Insoluble material was removed by centrifugation (5,000 g for 10 min at 4 °C) and the supernatant was loaded on the column of Sephadex G-50 (2.5×150 cm) equilibrated with 0.2 M ammonium acetate pH 7.1 and flow rate adjusted to 1 ml/min. Finally, protein absorbance was measured at 280 nm by UV spectrophotometer and four fractions were obtained (18).

*Mitochondrial Isolation from human EOC tissue*

For mitochondrial isolation, the human EOC tissue samples were collected after the surgery and were washed with ice-cold buffer consisting sucrose 0.25 M, Tris-HCl 5 mM, pH 7.4, and EDTA 1 mM, then minced and homogenized with glass handheld homogenizer in the chilled buffer. The homogenate was centrifuged at 2000×g for 10 min to discard the pellet the nuclei, unbroken cells, and tissue debris. The supernatant obtained from the first step was collected and then centrifuged at 14,000×g for 10 min. The pellet contains the purified mitochondria and then the extracted mitochondria were suspended in cold buffer. All the steps were performed at 4 °C (Upadhyay *et al*., 2002). The protein concentrations were determined using the Bradford assay through the Coomassie blue protein-binding method using BSA as the standard (19).

*Determination of succinate dehydrogenase activity*

Briefly, the effect of crude extract and its fractions of *T. coronatus* (0, 50, 100, 200, 500 and 1000 μg/mL) on SDH activity were measured by the decline of MTT (3-[4, 5-dimethylthiazol-2-yl]-2, 5-diphenyltetrazolium bromide)to formazan using an ELISA reader (Tecan, Rainbow Thermo, Austria) at 570 nm (20). 

*Determination of mitochondrial ROS formation*

We used the DCFH-DA as a fluorescent probe to assess the mitochondrial reactive oxygen species (ROS) level. The mitochondria isolated from cancerous and non-cancerous tissues were placed in the respiration buffer. The fluorescent probe at concentration of 10 μM was added to mitochondria isolated from both groups and incubated for 10 min at 37 °C. Finally, mitochondrial fluorescence (DCF) as an indicator of the level of ROS generation was measured by Shimadzu RF-5000U fluorescence spectrophotometer (Ex: 488nm and EM: 527nm) (21). 

*Determination of mitochondrial membrane potential (MMP)*

The mitochondrial isolated from cancerous and non-cancerous tissues were incubated with 10 mM rhodamine 123 in MMP assay buffer and then various concentrations of F1 fraction of *T. coronatus *crude extract was added for1 h. The fluorescence activity was monitored at the excitation and emission wavelength of 490nm and 535 nm respectively, by using fluorescence spectrophotometer (Schimadzou RF-5,000U) (22).

*Determination of mitochondrial swelling*

The effect of F1 fractions of *T. coronatus *crude extract in mitochondrial swelling was measured spectrophotometrically at 5, 15, 30, 45, and 60 min time intervals using an ELISA reader (Tecan, Rainbow Thermo, Austria). Mitochondrial swelling results in a decrease in absorbance monitored at 540 nm (23).

*Determination of cytochrome c releases*

The amount of cytochrome c released to the medium from the isolated mitochondria in response to various concentrations of F1 fraction of *T. coronatus* was determined at 450 nm according to the instructions provided by the manufacturer of the Quantikines Rat/Mouse Cytochrome c Immunoassay Kit (R&D Systems, Inc., Minneapolis, USA). 

*Isolation of primary ovarian cancer cells from tumoral specimens*

The solid specimens of ovarian cancer were washed in 10 mL of fresh, ice cold phosphate-buffered saline (PBS) then with sterile razor blade cut into to the smallest sections. Minced tissues were transferred into a 15 mL tube containing 10 ml DMEM (Dulbecco’s modified Eagle medium) with 0.1% collagenase and incubated at 37 °C for 30 min. After 30 min incubation, the cell suspension was centrifuged at 300×g for 4 min at 4 °C. Finally, the cell suspensions were plated in DMEM containing 10% FBS and supplemented with 100 U /mL penicillin, 100 µg /mL streptomycin, 2 mM glutamine, 10 mM Hepes (24).

*Determination of Cytotoxicity by MTT assay*

The effect of the F1 fraction of *T. coronatus* crude extract on cancerous and non-cancerous cells from patients with EOC was assessed using the MTT assay(25).Briefly, the cells (1×10^6^cells/mL) were plated in 96-well culture plates. The F1 fraction of *T. coronatus* crude extract was added to various final concentrations (0-1000 μg/mL) in triplicates. After 12 h of incubation with F1 fraction (37 °C and 5% CO_2_), 25 mL of MTT reaction solution (5 mg/ mL) was added to the wells and incubated for an additional 4 h. The purple-blue MTT formazan precipitate was dissolved in 100 μL of DMSO, and the absorbance was measured at 570 nm wavelength with an ELISA reader.

*Determination of Caspase-3 Activity*

The effect of the crude extract and F1 fraction on the activation of caspase-3 in cancerous and non-cancerous cells of EOC were assessed according to the instructions of the Sigma’s caspase-3 colorimetric assay kit (CASP-3-C; Sigma-Aldrich, Saint Louis, USA). Eventually, the concentration of the p-nitroaniline released from the substrate peptide, Ac- DEVD-pNA at 405 nm was used for evaluate caspase- 3 activity.

*Detection of Apoptosis versus Necrosis by Propidium Iodide*

Cancerous and non-cancerous cells (1×10^6^cells/mL)from patients with EOC exposed to F1 fraction and apoptosis versus necrosis % were evaluated at 12h by flow cytometric double staining analysis (Cyflow Space-Partec) of Annexin V-fluorescein isothiocyanate (FITC)/propidium iodide (PI) stained cells by using a commercial kit (Immunotech; Beckman Coulter)(26).

*Statistical analysis*

Statistical assays were performed using the GraphPad Prism software (version 6). The data were showed as mean ± SD and the assays were performed in triplicate. Comparisons were made using the one-way (followed by the post-hoc Tukey test) and two-way (Bonferroni post-hoc test) ANOVA tests. *P *< 0.05 was considered as level of significance.

## Results

*Gel filtration chromatography*

**Figure 1:**
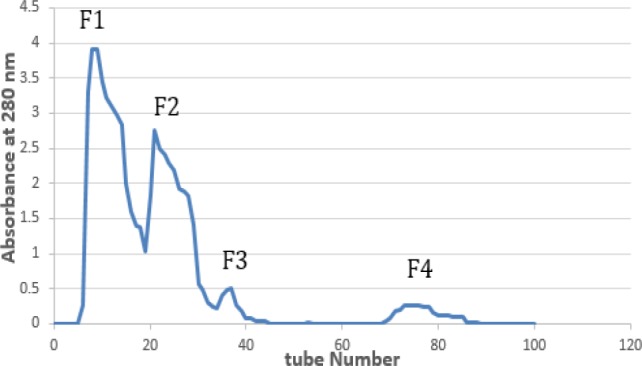
Gel filtration chromatogram of *Turbo coronatus* crude extract on G-50 (2.5×150 cm)

*T. coronatus* crude extract chromatogram on Sephadex G-50 showed four obvious peaks (F1-F4) based on their molecular weight with absorbance at 280 nm as shown in Figure 1.

*Mitochondrial Assays*

*Succinate Dehydrogenase Activity*

**Figure 2:**
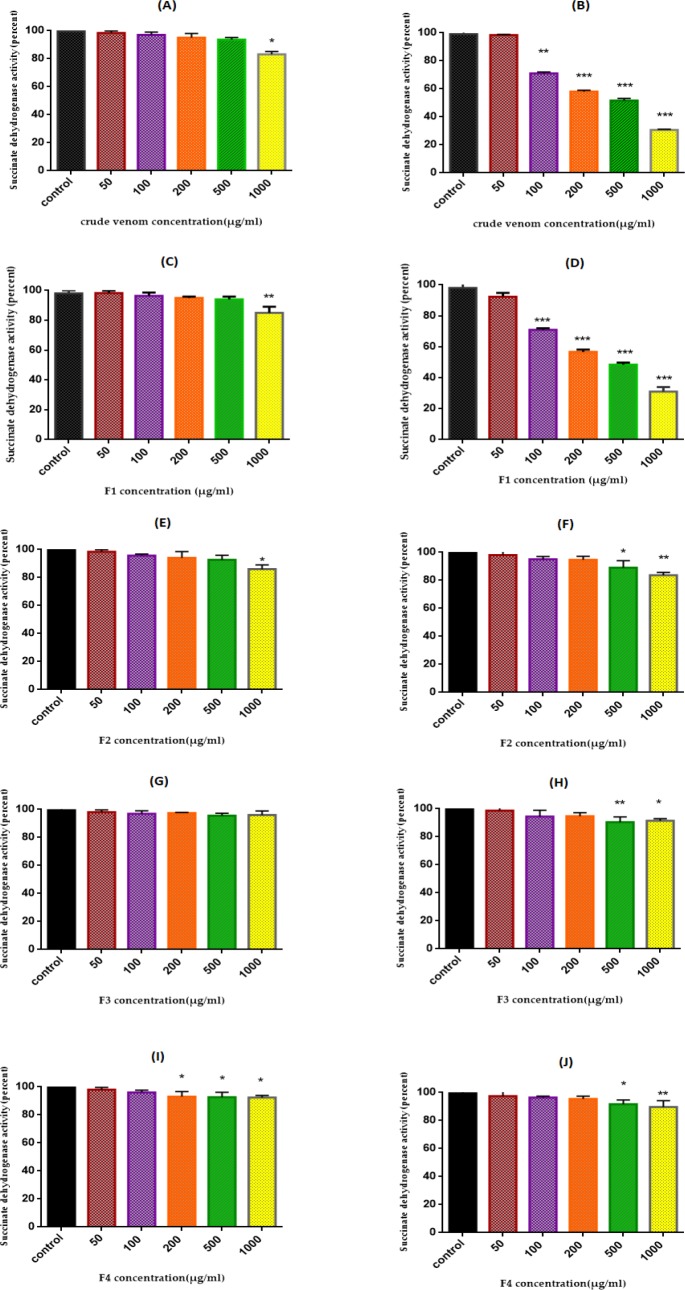
SDH Activity. Effect of various concentrations (0-1000 µg/mL) of crude extract and fractions (F1-F4) of *T. coronatus *extract on mitochondrial succinate dehydrogenase activity measured by MTT assay after 12 h treatment. A (non-cancerous plus crude extract); B (EOC plus crude extract); C (non-cancerous plus F1 fraction); D (EOC plus F1 fraction); E (non-cancerous plus F2 fraction); F (EOC plus F2 fraction); G (non-cancerous plus F3 fraction); H (EOC plus F3 fraction); I (non-cancerous plus F4 fraction); J (EOC plus F4 fraction). Data are shown as mean ± SD (n = 5). *, **, *** and **** show a significant difference in comparison with the corresponding control (*P *< 0.05, *P *< 0.01, *P *< 0.001 and *P *< 0.0001, respectively).

We examined the effect of *T. coronatus* crude extract and its fractions (0, 50, 100, 200, 500, and 1000 μg/mL) on isolated ovarian mitochondria obtained from both cancerous and non-cancerous tissues after 60 min of exposure with the MTT assay. Our results demonstrated thatF1 fraction signiﬁcantly (*P *< 0.001) reduced activity of SDH in the cancerous mitochondria but not in the normal mitochondria except at high concentration (1000 μg/mL) (Figure 2C and D). Finally, the measured IC_50_ for F1 fraction was 220 μg/mL. Based on the calculated IC_50_ the concentrations of 110, 220, and 440 μg/mL from F1 fraction were selected to determine other mitochondrial toxicity parameters.

*Mitochondrial ROS*

**Figure 3:**
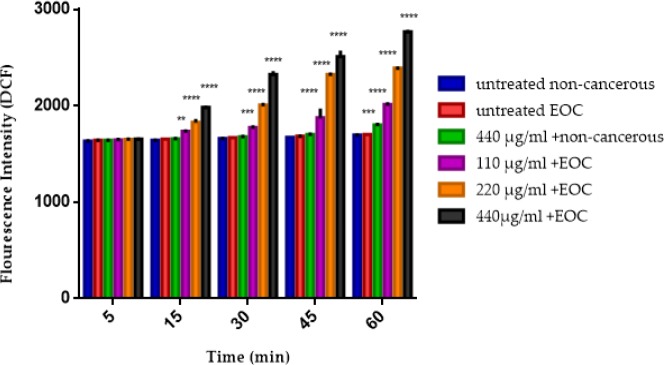
The mitochondrial ROS assay. The effect of various concentrations of F1 fraction (110, 220 and 440 µg/mL) of *T. coronatus* extract on mitochondrial ROS formation isolated from non-cancerous and EOC tissues. Data are shown as mean ± SD (n = 5). **, *** and **** show a significant difference in comparison with the untreated non-cancerous mitochondria (*P *< 0.01, *P *< 0.001 and *P *< 0.0001, respectively).

As shown in figure 3, F1 fraction at all applied concentrations induced mitochondrial ROS formation in the mitochondria isolated from the EOC but not in non-cancerous tissue except at the highest concentration of F1 fraction (440 μg) in the 60 min.

*Mitochondrial membrane potential*

**Figure 4:**
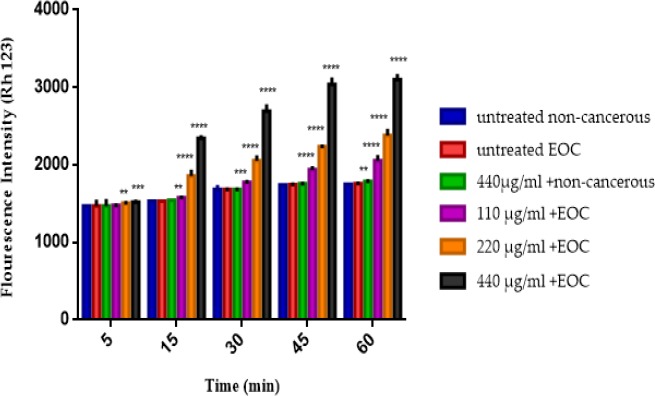
The MMP assay. The effect of various concentrations of F1 fraction (110, 220 and 440 µg/mL) of *T. coronatus *extract on decline of the MMP on the mitochondria obtained from non-cancerous and EOC tissues. Data are shown as mean ± SD (n = 5). **, *** And **** shows a significant difference in comparison with the untreated non-cancerous mitochondria (*P *< 0.01, *P *< 0.001 and *P *< 0.0001), respectively

As shown in Figure 4, different concentrations of F1 fraction of *T. coronatus* (110, 220 and 440 μg/mL) significantly (*P *< 0.05) decreased the MMP (demonstrated as fluorescence intensity units emitted from Rh123) only in mitochondria obtained from cancerous epithelial ovarian tissue but not in non-cancerous mitochondria except at a concentration of 440 μg in the 60 min.

*Mitochondrial Swelling*

**Figure 5:**
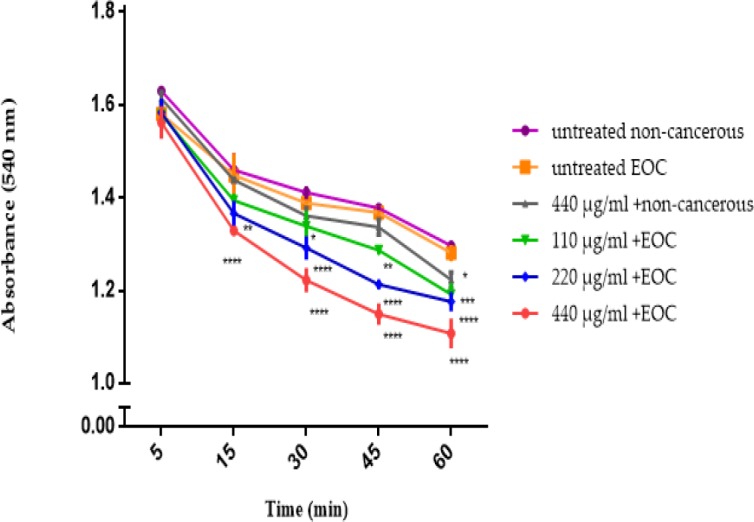
Mitochondrial Swelling Assay. Effect of various concentrations of F1 fraction of *T. coronatus *extract (110, 220 and 440 µg/ mL) of on mitochondrial swelling measured by decrease of mitochondrial suspensions absorbance in both cancerous and non-cancerous mitochondria. Data are shown as mean ± SD (n = 5). *, **, *** and **** shows a significant difference in comparison with the untreated non-cancerous mitochondria (*P *< 0.05, *P *< 0.01, *P *< 0.001 and *P *< 0.0001, respectively)

F1 fraction of *T. coronatus* (110, 220 and 440 μg/mL), induced swelling (decrease in absorption) in cancerous mitochondria but not in non-cancerous mitochondria at 540 nm. (Figure 5).

*Cytochrome c Release*

**Figure 6:**
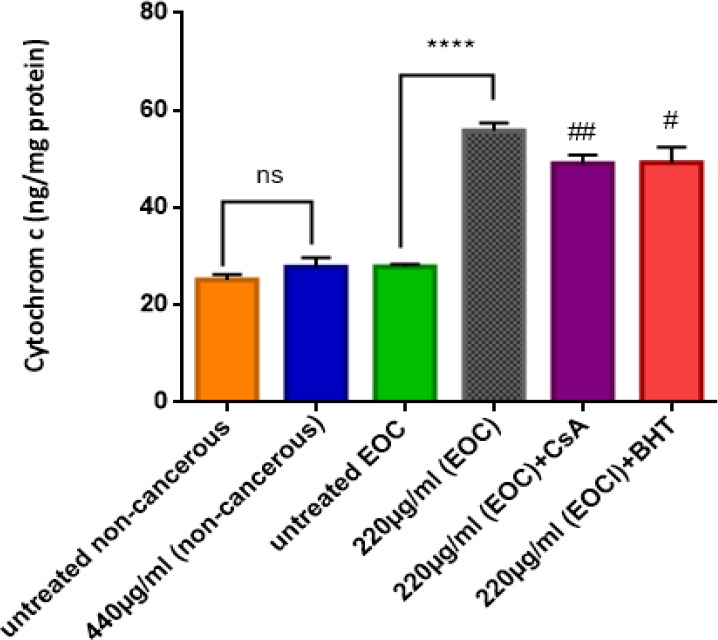
Cytochrome C Release Assay. The effect of IC50 concentration of F1 fraction (220 µg/mL) of *T. coronatus *on the cytochrome C release in cancerous and non-cancerous mitochondria. Data are shown as mean ± SD (n = 5). **** shows a significant difference in comparison with the untreated EOC (*P*<0.0001). # and ## show significant difference in comparison with F1 (440 µg/mL)-treated EOC mitochondria, (*P *< 0.05 and *P *< 0.001)

Our results demonstratethatF1 fraction at IC_50_ concentration (220 µg/mL) induced a significant (*P* < 0.05) release of cytochrome c only in the mitochondria obtained from cancerous epithelial ovarian tissue (Figure 6).Furthermore, pre-treatment of cancerous mitochondria with cyclosporine A (CsA); as a MPT inhibitors; and butylated hydroxyl toluene (BHT); as an antioxidant, significantly inhibited the induction of cytochrome c release by F1 fraction.

**Figure 7:**
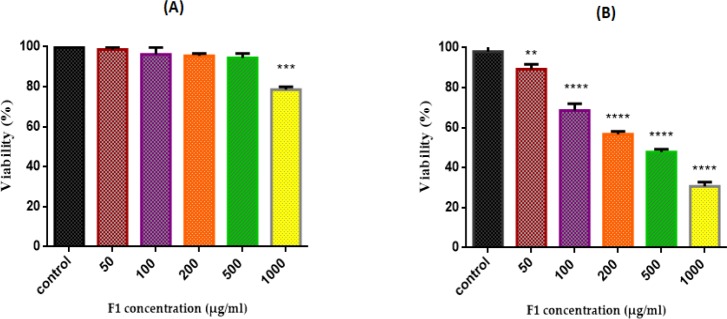
Effect of F1 fraction of *T. coronatus *extract concentration (0-1000 µg /mL) on cell viability in both EOC (A) non-cancerous(B) cells. Cells were treated with F1 and cell viability was measured by MTT Assay Following 12 h of exposure. Values are presented as mean ± SD (n = 5). **, *** and **** shows a significant difference in comparison with the untreated cancerous and non-cancerous cells (*P *< 0.01, *P *< 0.001 and *P *< 0.0001, respectively)

**Figure 8:**
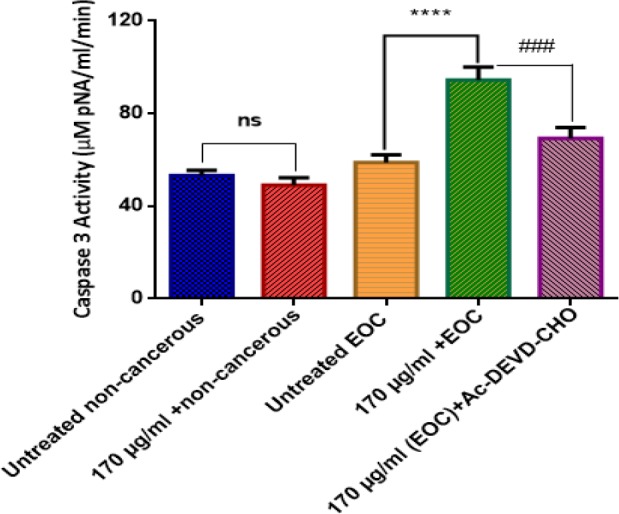
Effect of F1 fraction of *T. coronatus*(170 μg/mL) on the activity of caspase-3 in EOC and non-cancerous cells. Values are presented as mean ± SD (n = 5). **** show significant difference in comparison with the untreated EOC cells (*P *< 0.0001). ### show significant differences in comparison with F1 (170 μg/mL)-treated EOC cells (*P *< 0.0001)

**Figure 9:**
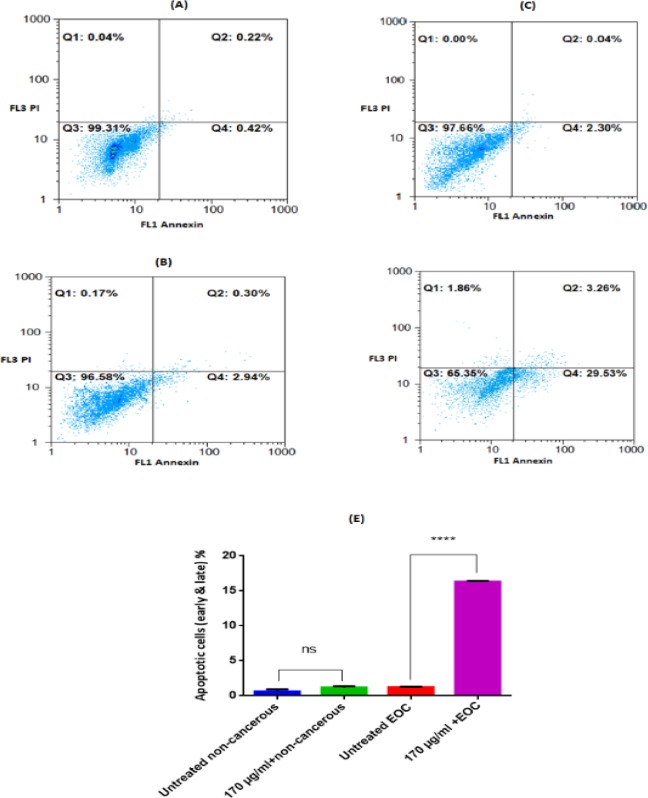
Analysis for Annexin-V and PI staining of non-cancerous and EOC Cells Incubated with F1 extract (170 μg/mL) for 12 h. Only Annexin V, positive (+) and PI, negative (−) cells were defined as apoptotic. A (untreated non-cancerous cells, Annexin V+/PI− = 0.42%), B (non-cancerous cells after 12h exposure of F1 fraction, Annexin V+/PI− = 2.94%), C (untreated EOC cells, Annexin V+/PI− = 2.30%), D (EOC cells after 12 h exposure of F1 fraction, Annexin V+/PI− = 29.53%). Q1: % necrotic cells, Q2: % late apoptotic cells, Q3: %Live cells, Q4: %early apoptotic cells. The summarized apoptotic data (early & later) was demonstrated at graph (E). Results are expressed as means ± SD (n = 5), **** *P *< 0.0001 vs. untreated non-cancerous cells

*Cellular Assays*

*Cell Viability Assay*

As shown in Figure 7 (A and B), the inhibiting effect of different concentrations of fraction F1 of *T. coronatus* (0, 50, 100, 200, 500 and 1000 μg/mL) on cell viability was determined with the MTT assay in cell suspensions (1×10^6^ cells/mL) isolated from both non-cancerous and cancerous epithelial ovarian tissues*.* However, F1 fraction significantly (*P *< 0.05) reduced cell viability only in cancerous but not non-cancerous cells. IC_50_ of F1 fraction treatment of cancerous cells was 170μg/mL.

*Caspase-3 Assay*

As shown in Figure 8, F1 fraction of *T. coronatus* crude extract significantly (*P < *0.05) increased the activity of caspase 3 as a final mediator of apoptosis only in ovarian epithelial cancer cells. On the other hand, our results showed that caspase3-induced activity by F1 fraction (170 μg/mL) was significantly reduced with pre-treatment of Ac-DEVD as a caspase-3 inhibitor in cancerous ovarian cells.

*Apoptosis and Necrosis*

As shown in Figure 9, apoptosis was quantiﬁed by the externalization of phosphatidylserine (PS), assessed by Annexin V-PI double staining at 12 h following the exposure with F1 fraction of *T. coronatus* (170 μg/mL). The percentage of apoptotic cells was significantly increased in cancerous ovarian cells by F1 fraction compared to the untreated cancer cells.

## Discussion

Various studies demonstrated that increased glycolysis in cancer cells occurs depending on the type and growth conditions of cancer cells in different degrees according to the Warburg Effect (27). Commonly, most of the required ATP of cancer cells is produced in the presence of oxygen through the aerobic glycolysis pathway, while normal cells often generate their ATP through mitochondrial oxidative phosphorylation using glucose, fatty acids, and other metabolic intermediates as energy sources. This increased dependence of cancer cells on the glycolytic pathway indicates significant mitochondrial dissimilarities between normal and cancerous cells and has been utilized for developing approaches and strategies to preferentially target cancerous mitochondria (28, 29). Recent studies reported that bioactive compound derived from marine mollusks trigger mitochondria to induce apoptosis in the cancer cell lines (30). So far, several studies have been carried out on various species of Turbo snails and have found compounds with cytotoxic, anti-cancer and pharmacological properties on different murine and human cell lines. These properties are attributed to their bioactive compounds (turbotoxin A and B; diiodotyramine, turbostatin 1-4; glycosphi ngolipid, taurine, etc.) (15, 16).The present results for the first time indicate that F1 fraction of *T. coronatus* crude extract induces ROS mediated apoptosis through the mitochondrial pathway in human EOC. F1 fraction of *T. coronatus* crude extract increased ROS production in a time and concentration dependent manner only in mitochondria isolated from cancerous tissue. Excessive production of ROS in different types of cancer cells can lead to oxidative stress induction (31). Increased oxidative stress reduces cell proliferation in many cancer cells including ovarian cancer. In another study, the accumulation of ROS by β-phenethylisothiocyanate (PEITC) induced apoptosis in ovarian cancer (32).Recently, several anticancer drugs have been shown to stimulate ROS generation from mitochondria in the cancer cells such as arsenic trioxide (As2O3), bleomycin, cisplatin, and anthracyclines (33, 34). Many documents indicate that reactive oxygen species induce mitochondrial membrane depolarization leading to organelles welling, mitochondrial permeability transition (MPT) pore-opening, and cytochrome cexpulsion from the mitochondria (35).Our data clearly showed that the applied concentrations of F1 fraction of *T. coronatus* crude extract caused significant MMP collapse in mitochondria isolated from EOC but not non-cancerous tissue. Following these experiment results, we monitored the mitochondrial swelling as an indicator of MPT. F1 Fraction of *T. coronatus* crude extract induced significant mitochondrial swelling again only in the mitochondria obtained from cancerous epithelial ovarian tissue but not non-cancerous tissue. Also, the release of cytochrome c as a subsequent event after MPT was assayed. This study shows the involvements of MPT pore opening in cytochrome c release from the cancerous mitochondria exposed to IC_50_ concentration (220 μg/mL) F1 fraction of *T. coronatus* crude extract. Moreover, pretreatment with both CsA as an inhibitor of MPT pore and BHT as a ROS scavenger significantly inhibited the cytochrome c release induced byF1 fraction. Eventually, we proved that F1 fraction of *T. coronatus* crude extract induces the increase of ROS production, collapse of the MMP, mitochondrial swelling, cytochrome c release, and increase caspase 3 activity in EOC cells. Quite similar to our present results, it was reported that the ethyl acetate extract from *Meretrixlusoria* could induce apoptosis in human leukemia cells via ROS production, glutathione depletion, and caspase activation(36). Our data is also in agreement with published work, which indicates an increase in caspase-3 activity by F1 fraction ref. In another study ona marine mollusk, bioactive compounds isolated from the egg mass extract of *Dicathaisorbita*, induced an apoptotic effect by increasing caspase 3/7 activity, and arresting the G2/M phase of the cell cycle in the colorectal cancer cell lines (37). In another report, the sepia ink oligopeptide extracted from marine mollusk *Sepia esculenta* induced apoptosis in prostate cancer cell lines through caspase-3 activation, upregulation of Bax protein expression, and downregulationin Bcl-2 level (38).The result obtained in our study shows that the F1 fraction of marine mollusk *Turbo coronatus *causes the induction of ROS mediated apoptosis via mitochondrial pathways in EOC cells and mitochondria. These findings are generally in accordance with the findings of other researchers with other species of marine mollusk in different cancer cell lines. Apoptotic cell death counteracts the tumor development of EOC. Therefore, in patients with ovarian cancer, the reduced Box expression is associated with poor response to the treatment. However, in the late stage of ovarian cancer, the anti-apoptotic protein of Bcl-2 was found to be over-expressed (39, 40). Thus, the induction of apoptosis can be a potential therapeutic strategy for treatment of EOC. In conclusion, the present study suggests that F1 fraction of *T. coronatus* crude extract with ignorable cytotoxicity towards non-cancerous ovarian cells may act as a promising anti-tumor agent against epithelial ovarian cancer cells by inducing apoptosis through ROS-mediated mitochondrial pathway.

## References

[1] Pashaei-Asl R, Pashaei-Asl F, Mostafa Gharabaghi P, Khodadadi K, Ebrahimi M, Ebrahimie E, Pashaiasl M (2017). The Inhibitory Effect of Ginger Extract on Ovarian Cancer Cell Line; Application of Systems. Biology. Adv. Pharm. Bull.

[2] Xu X-H, Liu Q-Y, Li T, Liu J-L, Chen X, Huang L, Qiang W-A, Chen X, Wang Y, Lin L-G, Lu J-J (2017). Garcinone E induces apoptosis and inhibits migration and invasion in ovarian cancer cells. Sci. Rep.

[3] Siegel RL, Miller KD, Jemal A (2018). Cancer statistics, 2018. CA Cancer. J. Clin.

[4] Desai A, Xu J, Aysola K, Qin Y, Okoli C, Hariprasad R, Chinemerem U, Gates C, Reddy A, Danner O, Franklin G, Ngozi A, Cantuaria G, Singh K, Grizzle W, Landen C, Partridge EE, Rice VM, Reddy ES, Rao VN (2014). Epithelial ovarian cancer: An overview. World J. Transl. Med.

[5] Choi CH, Ryu JY, Cho YJ, Jeon HK, Choi JJ, Ylaya K, Lee YY, Kim TJ, Chung JY, Hewitt SM, Kim BG, Bae DS, Lee JW (2017). The anti-cancer effects of itraconazole in epithelial ovarian cancer. Sci. Rep.

[6] Chowdhury SR, Ray U, Chatterjee BP, Roy SS (2017). Targeted apoptosis in ovarian cancer cells through mitochondrial dysfunction in response to Sambucus nigra agglutinin. Cell Death. Dis.

[7] Koff JL, Ramachandiran S, Bernal-Mizrachi L (2015). A Time to Kill: Targeting Apoptosis in Cancer. Int. J. Mol. Sci.

[8] Hsu CC, Tseng LM, Lee HC (2016). Role of mitochondrial dysfunction in cancer progression. Exp. Biol. Med (Maywood).

[9] Cragg GM, Pezzuto JM (2016). Natural Products as a Vital Source for the Discovery of Cancer Chemotherapeutic and Chemopreventive Agents. Med. Princ. Pract.

[10] Mann J (2002). Natural products in cancer chemotherapy: past, present and future. Nat. Rev. Cancer.

[11] Ruiz-Torres V, Encinar JA, Herranz-Lopez M, Perez-Sanchez A, Galiano V, Barrajon-Catalan E, Micol V (2017). An Updated Review on Marine Anticancer Compounds: The Use of Virtual Screening for the Discovery of Small-Molecule Cancer Drugs. Molecules.

[12] Blunt G, Copp B, keyzers R, Munro M, Princep M (2016). Marine natural products. Nat. Prod. Rep.

[13] Honari M, Tehranifard A, Vazirian M, Salaritabar A, Sanati H, Ansari MA (2017). Cytotoxic Effect of Turbo coronatus Extract on Cancer Cell Lines. Research Reviews: Pharmacy & Pharmaceutical Sciences.

[14] Lasiak T (1986). The reproductive cycle of the intertidal gastropod Turbo coronatus Gnlelin 1791, on the Transkei coast. African Zoology.

[15] Pettit GR, Tang Y, Knight JC (2005). Antineoplastic Agents 545 Isolation and Structure of Turbostatins 1–4 from the Asian Marine Mollusc Turbo stenogyrus(,1). J. Nat. Prod.

[16] Kigoshi H, Kanematsu K, Uemura D (1999). Turbotoxins A and B, novel diiodotyramine derivatives from the Japanese gastropod Turbo marmorata. Tetrahedron Lett.

[17] Vazirizadeh A, Mohebbi G, Nabipour I (2017). The Effect of Crude Extract of Turbo coronatus from the Persian Gulf on Serum Biochemical Parameters and Hematiological Parameters of Rats. BPUMS.

[18] Samel M, Tonismagi K, Ronnholm G, Vija H, Siigur J, Kalkkinen N, Siigur E (2008). L-Amino acid oxidase from Naja naja oxiana venom. Comp. Biochem. Physiol. B Biochem. Mol. Biol.

[19] Hosseini MJ, Shaki F, Ghazi-Khansari M, Pourahmad J (2013). Toxicity of vanadium on isolated rat liver mitochondria: a new mechanistic approach. Metallomics.

[20] Mirshamsi MR, Omranipour R, Vazirizadeh A, Fakhri A, Zangeneh F, Mohebbi GH, Seyedian R, Pourahmad J (2017). Persian Gulf Jellyfish (Cassiopea andromeda) Venom Fractions Induce Selective Injury and Cytochrome C Release in Mitochondria Obtained from Breast Adenocarcinoma Patients. Asian Pac. J. Cancer Prev.

[21] Salimi A, Roudkenar MH, Sadeghi L, Mohseni A, Seydi E, Pirahmadi N, Pourahmad J (2015). Ellagic acid, a polyphenolic compound, selectively induces ROS-mediated apoptosis in cancerous B-lymphocytes of CLL patients by directly targeting mitochondria. Redox Biol.

[22] Rezaei M, Salimi A, Taghidust M, Naserzadeh P, Goudarzi GR, Seydi E, Pourahmad J (2014). A comparison of toxicity mechanisms of dust storm particles collected in the southwest of Iran on lung and skin using isolated mitochondria. Toxicol. Environ. Chem.

[23] Talari M, Seydi E, Salimi A, Mohsenifar Z, Kamalinejad M, Pourahmad J (2014). Dracocephalum: novel anticancer plant acting on liver cancer cell mitochondria. Biomed. Res. Int.

[24] Pribyl LJ, Coughlin KA, Sueblinvong T, Shields K, Iizuka Y, Downs LS, Ghebre RG, Bazzaro M (2014). Method for Obtaining Primary Ovarian Cancer Cells From Solid Specimens. J. Vis. Exp.

[25] Zhang Y, Luo M, Zu Y, Fu Y, Gu C, Wang W, Yao L, Efferth T (2012). Dryofragin, a phloroglucinol derivative, induces apoptosis in human breast cancer MCF-7 cells through ROS-mediated mitochondrial pathway. Chem. Biol. Interact.

[26] Qiu LN, Zhou YL, Wang ZN, Huang Q, Hu WX (2012). ZGDHu-1 promotes apoptosis of chronic lymphocytic leukemia cells. Int. J. Oncol.

[27] Theodosakis N, Micevic G, Kelly DP, Bosenberg M (2014). Mitochondrial function in melanoma. Arch. Biochem. Biophys.

[28] Gogvadze V, Zhivotovsky B, Orrenius S (2010). The Warburg effect and mitochondrial stability in cancer cells. Mol. Aspects Med.

[29] Upadhyay M, Samal J, Kandpal M, Singh OV, Vivekanandan P (2013). The Warburg effect: insights from the past decade. Pharmacol. Ther.

[30] Ciavatta ML, Lefranc F, Carbone M, Mollo E, Gavagnin M, Betancourt T, Dasari R, Kornienko A, Kiss R (2017). Marine Mollusk-Derived Agents with Antiproliferative Activity as Promising Anticancer Agents to Overcome Chemotherapy Resistance. Med. Res. Rev.

[31] Simon HU, Haj-Yehia A, Levi-Schaffer F (2000). Role of reactive oxygen species (ROS) in apoptosis induction. Apoptosis.

[32] Hong YH, Uddin MH, Jo U, Kim B, Song J, Suh DH, Kim HS, Song YS (2015). ROS Accumulation by PEITC Selectively Kills Ovarian Cancer Cells via UPR-Mediated Apoptosis. Front Oncol.

[33] Arast Y, Seyed Razi N, Nazemi M, Seydi E, Pourahmad J (2018). Non-polar compounds of Persian Gulf sea cucumber Holothuria parva selectively induce toxicity on skin mitochondria isolated from animal model of melanoma. Cutan. Ocul. Toxicol.

[34] Seydi E, Rasekh HR, Salimi A, Mohsenifar Z, Pourahmad J (2016). Myricetin Selectively Induces Apoptosis on Cancerous Hepatocytes by Directly Targeting Their Mitochondria. Basic Clin. Pharmacol. Toxicol.

[35] Mirshamsi M, Vazirizadeh A, Omranipour R, Fakhri A, Aghvami M, Naserzadeh P, Zangeneh F, Pourahmad J (2017). Persian Gulf Stonefish (Pseudosynanceia melanostigma) Venom Fractions Selectively Induce Apoptosis on Cancerous Hepatocytes from Hepatocellular Carcinoma Through ROS-Mediated Mitochondrial Pathway. Hepat. Mon.

[36] Pan MH, Huang YT, Ho CT, Chang CI, Hsu PC, Sun Pan B (2006). Induction of apoptosis by Meretrix lusoria through reactive oxygen species production, glutathione depletion, and caspase activation in human leukemia cells. Life Sci.

[37] Esmaeelian B, Benkendorff K, Johnston MR, Abbott CA (2013). Purified brominated indole derivatives from Dicathais orbita induce apoptosis and cell cycle arrest in colorectal cancer cell lines. Mar. Drugs.

[38] Huang F, Yang Z, Yu D, Wang J, Li R, Ding G (2012). Sepia ink oligopeptide induces apoptosis in prostate cancer cell lines via caspase-3 activation and elevation of Bax/Bcl-2 ratio. Mar. Drugs.

[39] Kar R, Sen S, Singh A, Sharma H, Kumar S, Gupta SD, Singh N (2007). Role of apoptotic regulators in human epithelial ovarian cancer. Cancer Biol. Ther.

[40] Cohen C, Lohmann CM, Cotsonis G, Lawson D, Santoianni R (2003). Survivin expression in ovarian carcinoma: correlation with apoptotic markers and prognosis. Mod. Pathol.

